# Moderate toxicity with late onset as a good omen: association between toxicity and survival in the checkpoint inhibitor immunotherapy—a single center experience

**DOI:** 10.3389/fimmu.2025.1527103

**Published:** 2025-04-28

**Authors:** Anna Rudzińska, Pola Juchaniuk, Jakub Oberda, Kamila Krukowska, Sylwia Krzyśkowska, Eliza Kuchta, Anna Rodzajewska, Mariola Janiszewska, Katarzyna Machulska-Ciuraj, Katarzyna Szklener

**Affiliations:** ^1^ Department of Clinical Oncology and Chemotherapy, Medical University of Lublin, Lublin, Poland; ^2^ Department of Medical Informatics and Statistics with e-Health Lab, Medical University of Lublin, Lublin, Poland

**Keywords:** cancer immunotherapy, immunotherapy toxicity, immune checkpoint inhibitors, immunotherapy adverse effects, immune check inhibitor (ICI)

## Abstract

Immune checkpoint inhibitors (ICIs) have revolutionized cancer therapy by enhancing T-cell-mediated immune responses against tumors. However, their use can lead to immune-related adverse events (irAEs) impacting patient outcomes. This single-center, observational study investigates the relationship between immune-related adverse events (irAEs) and survival outcomes and, to our knowledge, is the first of this kind in Polish population. Data of the 151 patients treated with ICIs, with or without chemotherapy, at the Department of Clinical Oncology and Chemotherapy in the Independent Public Hospital No. 4 in Lublin were collected from electronic medical records. Statistical analyses were performed using the Kaplan–Meier estimator, log-rank test, and multivariable Cox proportional hazard model (p < 0.05). IrAEs were observed in 38% of the patients, with the most common being thyroid dysfunction (11.9%) and dermal toxicity (6.6%). The median OS for patients with irAEs was 18.7 months, compared to 13.6 months for those without irAEs, though the difference was not statistically significant (p = 0.284). Patients with moderate toxicity had the highest median OS (26 months), while those with severe toxicity had a median OS of 6.41 months. Late-onset irAEs were associated with improved OS and PFS. Pack-years of smoking significantly impacted both OS (*HR* = 1.01, p = 0.014) and PFS (*HR* = 1.01, p = 0.011). Despite results not reaching statistical significance, the findings emphasize the clinical relevance of irAEs in treatment optimization and warrant further research to better understand their role in patient outcomes.

## 1 Introduction

The discovery of immunological checkpoints has revolutionized the treatment of cancer. Among many control points used by tumors to avoid host immune responses, the best known and most commonly used in clinical practice are PD-1/PD-L1 and CTLA-4 pathways (1–3). Immunomodulatory therapies in lung cancer block inhibitory signals between cancer cells and T cells allowing T cells to recognize and attack the tumor (4).

Immune checkpoint inhibitors (ICI) have been approved for the use in the treatment of multiple cancer types, including melanoma, non-small-cell lung cancer (NSCLC), urothelial cancer, cancer of the head and neck, and uterine cancer (5). Pharmaceutics, such as nivolumab, pembrolizumab, and atezolizumab, have shown particular relevance in improving survival rates in patients with advanced lung and urothelial cancer (6, 7).

The specific mechanism of action of ICIs influencing the lymphocytic response can lead to nonspecific activation of the immune system and development of autoimmune-like diseases as side effects of treatment (5). Diarrhea, nausea, thyroid dysfunction, and rash belong to the most common adverse effects of cancer immunotherapy, which can be either a result of immunomodulation or treatment intolerance (7). Checkpoint inhibitors can affect the immune function of many organs and systems (5). Immune-related adverse events (irAEs) can manifest as colitis, pituitary inflammation, pneumonia, thyroiditis, or fatigue, among others (3).

The overall incidence of irAEs has been low in clinical trials evaluating monotherapy with anti-PD-1 and anti-PD-L1 therapies, usually reaching <5% (3). Common Terminology Criteria for Adverse Events (CTCAE) is used to assess and classify irAEs (8). The influence of the occurrence of the irAE on the further course of the clinical management, treatment regime, response rate, and survivability became, in recent years, the topic of great interest (3, 5). In our study we investigated if the occurrence and intensity of the irAE during the course of the immunotherapy could be linked to the survivability of the treated patients. To our knowledge, this is the first study of said link in the Polish population.

## 2 Patients and methods

This single-center, observational study was conducted using medical records of 151 patients with any solid tumor treated with at least one dose of immunotherapy with or without chemotherapy at the Department of Clinical Oncology and Chemotherapy in the Independent Public Hospital No. 4 in Lublin between 2019 and 2023. Data cut-off date was 26 April 2023. All examined patients were adults (>18 years old), with varying cancer subtypes—predominantly NSCLC. PD-L1 expression detected by the SP263 antibodies in 97 patients varied between 0% and 100%. In the remaining patients, PD-L1 expression scores were unattainable. Immunotherapy administered included pembrolizumab, nivolumab, atezolizumab, avelumab, durvalumab, and ipilimumab in addition to PD-1 inhibitor. Chemotherapy regimens consisted of platinum in combination with other drugs, such as pemetrexed, docetaxel, gemcitabine, or paclitaxel/nab-paclitaxel. We collected patient baseline clinical data through electronic medical records, including age, sex, cancer stage, histology, differentiation, smoking history including smoking status and pack years, TNM classification, line of therapy, treatment type, clinical response, time of onset of the irAEs, type of the irAEs (organ-specific), grade of the irAEs, overall survival and progression-free survival.

Patients’ irAEs were defined based on pathological proof, laboratory results,and clinician decision after excluding other causes. Toxicities were graded by physicians based on Common Terminology for Adverse Events criteria v4.0 (CTCAE v4.0). In patients receiving immunotherapy with chemotherapy or who received chemotherapy as a previous treatment line, we distinguished between immunotherapy- and chemotherapy-related adverse events based on the differences in the toxicity spectrum (incidence rate and treatment-specific adverse effects) and the time of toxicity onset. Hematological disorders and neuropathy were the most excluded adverse effects. As the data collection relied on the medical records available, it is possible that the first- and second-grade adverse events are underrepresented in our study. It might have been caused by inaccurate reporting of the physicians, as the lower-grade toxicities are often times misdiagnosed or underreported in the medical documentation due to their mild nature and minimal interference in the therapeutic process.

Tumor response was evaluated by the tomography scan results using the RECIST (Response Evaluation Criteria in Solid Tumors) 1.1 criteria. PFS was defined as the time from the first administration of the immunotherapy until disease progression, unacceptable toxicity resulting in a change of treatment line, death, or follow-up cut-off date. OS was defined as the time from the first day of ICI treatment administration of the immunotherapy until death or follow-up cut-off date. This study was approved by the Medical University of Lublin institutional review board (No. KE-0254/198/10/2022).

Data distribution was tested for normality with the Shapiro–Wilk test. The irregularity of the distribution allowed for the utilization of non-parametric statistical methods for the analyzed variables. The analysis of survival function from the beginning of therapy until the occurrence of the events (deaths and progressions) was conducted using the Kaplan–Meier estimator. Additionally, to complement the analysis of survival graphs, risk tables were implemented providing a numerical perspective on the data.

To compare the survival time between groups, the log-rank test was used. In the context of analyzing the impact of multiple factors on the survival time, the multivariable Cox proportional hazard model was used. The significance of the model and individual variables was investigated by the following tests: likelihood ratio test, Wald, and score. Before using the Cox model, the proportional hazard test was evaluated.

The effect size for individual variables was expressed using the hazard ratio (HR). The multivariable Cox proportional hazard model fitting was rated by using the *R²_Nagelkerke_* measure, that provides information about the proportion of variances in survival times explained by the models.

For assessing the association between two continuous variables, the Spearman’s rank correlation coefficient was conducted. The statistical significance of the correlation coefficient was computed using an asymptotic approximation of the distribution *t*.

The significance of differences between two or more groups with non-normal distribution was estimated with the ANOVA Kruskal–Wallis test. In terms of effect size, the measure of epsilon squared was calculated. All statistical analysis results were presented with an adequate significance level (α = 0.050), which enabled the assessment of the credibility of the observed relationships and conclusions drawn from the study.

The analysis was performed using the statistic language R (version 4.3.1; R Core Team, 2023), in Windows 10 pro 64 bit system (compilation 19045), using the *car* packages (version 3.1.2; Fox J, Weisberg S, 2019), *sjPlot* (version 2.8.15; Lüdecke D, 2023), *parameters* (version 0.21.3; Lüdecke D et al., 2020), performance (version 0.10.8; Lüdecke D et al., 2021), report (version 0.5.7; Makowski D et al., 2023), *ggsurvfit* (version 1.0.0; Sjoberg D et al., 2023), *gtsummary* (version 1.7.2; Sjoberg D et al., 2021), *survival* (version 3.5.5; Therneau T, 2023), *ggplot2* (version 3.4.4; Wickham H, 2016), *readxl* (version 1.4.3; Wickham H, Bryan J, 2023), and *dplyr* (version 1.1.3; Wickham H et al., 2023).

## 3 Results

In the study group among patients with cancer, the median age was 69.0 years. Women represented 35.1% (*n* = 53) and men 64.9% (*n* = 98) of the cohort. Ex-smokers constituted nearly 41% and active smokers 26.5% of the group. Non-smokers represented almost one-third of the cohort (32.5%). The median pack-years was 20. The median PD-L1 expression count reached 20%. PD-L1 expression was negatively correlated to the pack-years parameter, although it did not reach statistical significance (p = 0.051). In most patients, immunotherapy constituted for the first (*n* = 69, 45.7%) or second (*n* = 77, 51.0%) line of the systemic treatment. Among 151 patients, most of them (65.6%, *n =* 99) were treated with the anti-PD-1 antibodies, while the other 34.4% (*n =* 52) with anti-PD-L1 antibodies.

Among 151 patients, the most common cancer type was the non-small-cell lung cancer (NSCLC), which constituted 78.1% of the cases (*n =* 118). Bladder cancer was the second most prevalent cancer type representing 9.3% of the population (*n =* 14). The analysis of 115 NSCLC patients disclosed that the most common diagnosed subtype was squamous cell carcinoma (48.7%, *n =* 56) and adenocarcinoma (43.5%, *n =* 50).

Survival status demonstrated that 49.0% the patients (*n* = 74) died, and 51.0% (*n* = *77*) were censored during the analysis meaning that they were still alive, or their tracking data were lost. Progression status revealed that 53.0% of the patients (*n* = 80) experienced progression of the disease, and 47.0% (*n* = 71) had no proof of the disease progression.

Most of the patients (63.6%) had no toxicity symptoms; mild symptoms occurred in 12.6% of the patients. Moderate (17.0%) and severe (6.0%) toxicity were found in 26 and 9 patients. Very severe toxicity was found in one patient (0.7%). The most common site-specific toxicity was thyroid toxicity (11.8% *n* = 18).

Adverse event rates were relatively similar in both anti-PD-L1 and anti-PD1 treatment groups, with no significant differences between them (all p > 0.05). The occurrence of toxicities was not strongly dependent on the type of tumor with statistically nonsignificant relationship between tumor type and the number of toxicity outbreaks (p = 0.471).

Characteristics of the study cohort are presented in **Table 1**.

**Table 1:** Demographic characteristics of the patient’s cohort.

Characteristics	*N*	Distribution^1^
Age (years)	151	69.0 (64.5. 73.0)*^2^*
Sex	151	
Female		53 (35.1%)
Male		98 (64.9%)
Smoking status	151	
Active smoker		40 (26.5%)
Non-smoker		49 (32.5%)
Previous smoker		62 (41.0%)
Pack-years	151	20.0 (0.0, 40.0)
PD-L1 expression (%)	97	20 (0-100)
Type of cancer	151	
NSCLC		118 (78.1%)
Urinary bladder cancer		14 (9.3%)
SCLC		9 (6.0%)
Kidney cancer		7 (4.6%)
Other		3 (2.0%)
NSCLC subtype	115	
Squamous cell carcinoma		56 (48.7%)
Adenocarcinoma		50 (43.5%)
NOS		3 (2.6%)
Pleomorphic cell carcinoma		1 (0.9%)
Large-cell carcinoma		5 (4.3%)
Cancer stage		
I		14 (9.3%)
II		22 (14.6%)
III		28 (18.5%)
IV		87 (57.6%)
Metastases status		
Yes		67 (44.4%)
No		45 (29.8%)
Unknown		39 (25.8%)
Treatment	151	
Anti-PD1		99 (65.6%)
Anti-PD-L1		52 (34.4%)
Immunotherapy therapeutic		
Atezolizumab		38 (25.2%)
Avelumab		10 (6.6%)
Durvalumab		4 (2.6%)
Nivolumab		28 (18.5%)
Pembrolizumab		71 (47%)
Immunotherapy monotherapy		106 (70.2%)
Immunotherapy plus chemotherapy		42 (27.8%)
Immunotherapy PD-1 plus CTLA-4		3 (2%)
Number of previous systemic treatment lines		
0		69 (45.7%)
1		77 (51%)
2 and more		5 (3.3%)
Overall survival (OS), weeks	151	35.6 (21.9. 55.1)
Progression-free survival (PFS), weeks	151	26.6 (13.4. 44.4)
Survival status	151	
Dead		74 (49.0%)
Censored		77 (51.0%)
Progression status	151	
Progression		80 (53.0%)
No progression		71 (47.0%)
Initial response		
Partial response		33 (21.9%)
Disease stabilization		69 (45.7%)
Progression		24 (15.9%)
Death		20 (13.2%)
Not reached		5 (3.3%)
Toxicity sites	151	
Skin toxicity		10 (6.6%)
Thyroid toxicity		18 (11.8%)
Hepatotoxicity		12 (7.9%)
Kidney toxicity		4 (2.6%)
Fatigue		3 (2.0%)
Arthritis		2 (1.3%)
Number of toxicity sites	151	
None		95 (62.9%)
1		43 (28.5%)
2		10 (6.6%)
3		2 (1.3%)
10		1 (0.7%)
Time of the toxicity onset, cycles	151	0.0 (0.0. 3.0)*^2^*
Time of the toxicity onset, weeks	151	0.0 (0.0. 7.5) *^2^*
Toxicity	151	
None		96 (63.6%)
Mild		19 (12.6%)
Moderate		26 (17.2%)
Severe		9 (6.0%)
Extremely severe		1 (0.7%)

^1^ *n* (%).

^2^ *Mdn* (*Q1*, *Q3*).

*N*, sample size; *n*, group size; *Mdn*, median; *Q1*, first quartile (25%); *Q3*, third quartile (75%).

### 3.1 Analysis of toxicity factors in overall survival

The log-rank test analysis of survival differences in different toxicity and no toxicity groups showed lack of statistical significance (p = 0.140).

In the no toxicity group, median survival accounted for 13.57 months, in the mild toxicity group for 18.66 months, and in the moderate toxicity group for 26.28 months. Patients with severe (and very severe) toxicity have the lowest median of survival—6.41 months, with half of the patients dying in the first 6 months (survival rate 50%).

Log-rank test analysis of differences in survival between patient groups with toxicity (without division into severity of the symptoms) and without toxicity in the analyzed cohort did not show statistically significance (p = 0.284).

From the patient survival with different toxicity levels analysis results, the log-rank test showed an value of p = 0.055, which is close to the materiality level, suggesting that differences in survival might be statistically significant. The difference is particularly noticeable in survival between mild/moderate toxicity level and severe (merged with very severe) course.

**Figure 1:**
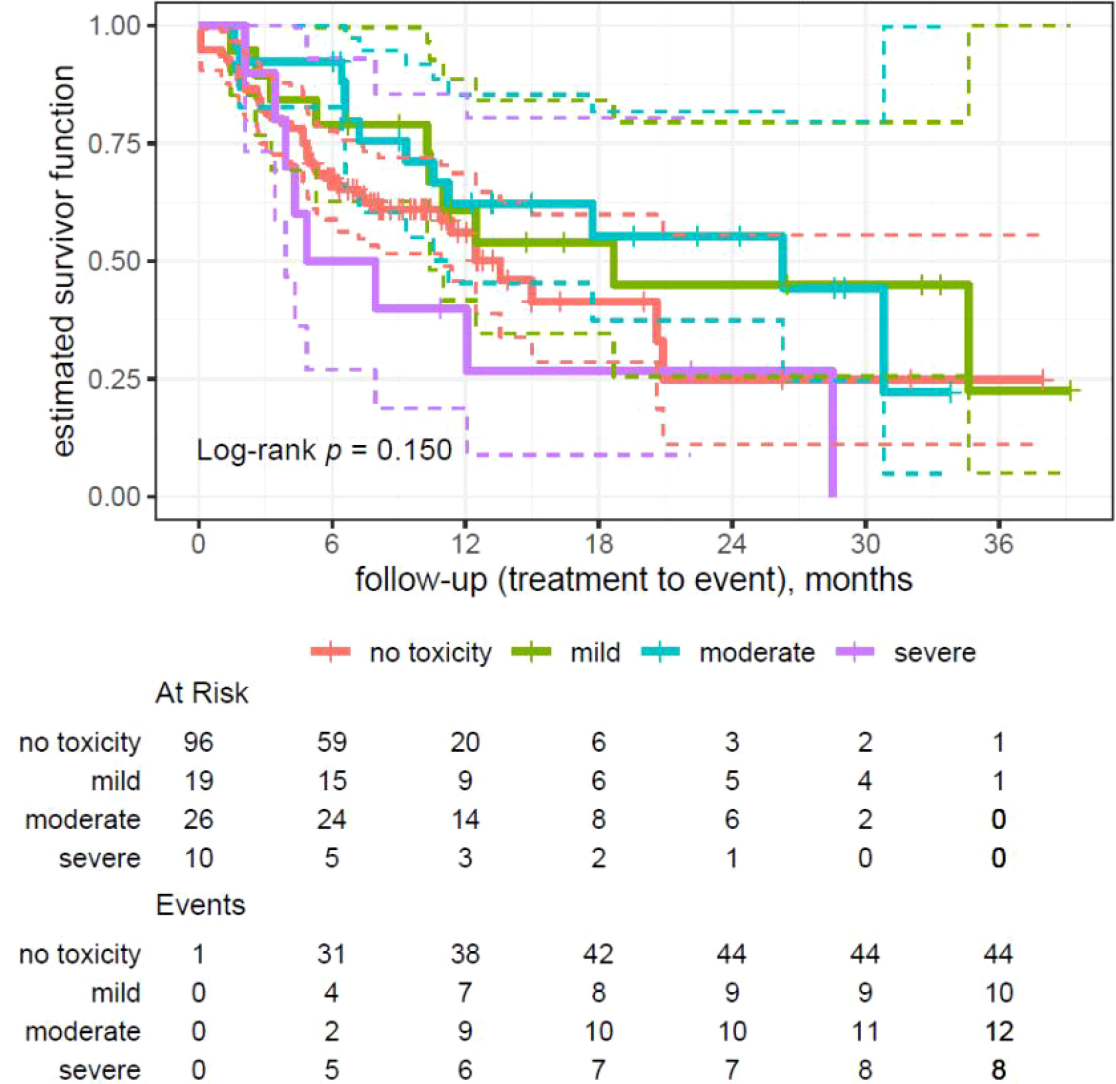
Kaplan–Meier analysis on patients’ overall survival divided by severity of toxicity(no toxicity, mild, moderate, and severe toxicity).

### 3.2 Analysis of toxicity factors in progression-free survival

Log-rank test (p = 0.400) did not show any statistically significant differences in PFS between the groups.

Patients with moderate toxicity have significantly longer PFS (median 26.28 months) in comparison to patients with mild (median 6.70 months) and severe toxicity (median 4.34 months). The group without toxicity presented a median PFS of 11.34 months.

The log-rank test did not show statistically significant differences in survival between patients with and without toxicity (p = 0.647) and in median PFS for different toxicity grades (p = 0.203).

**Figure 2:**
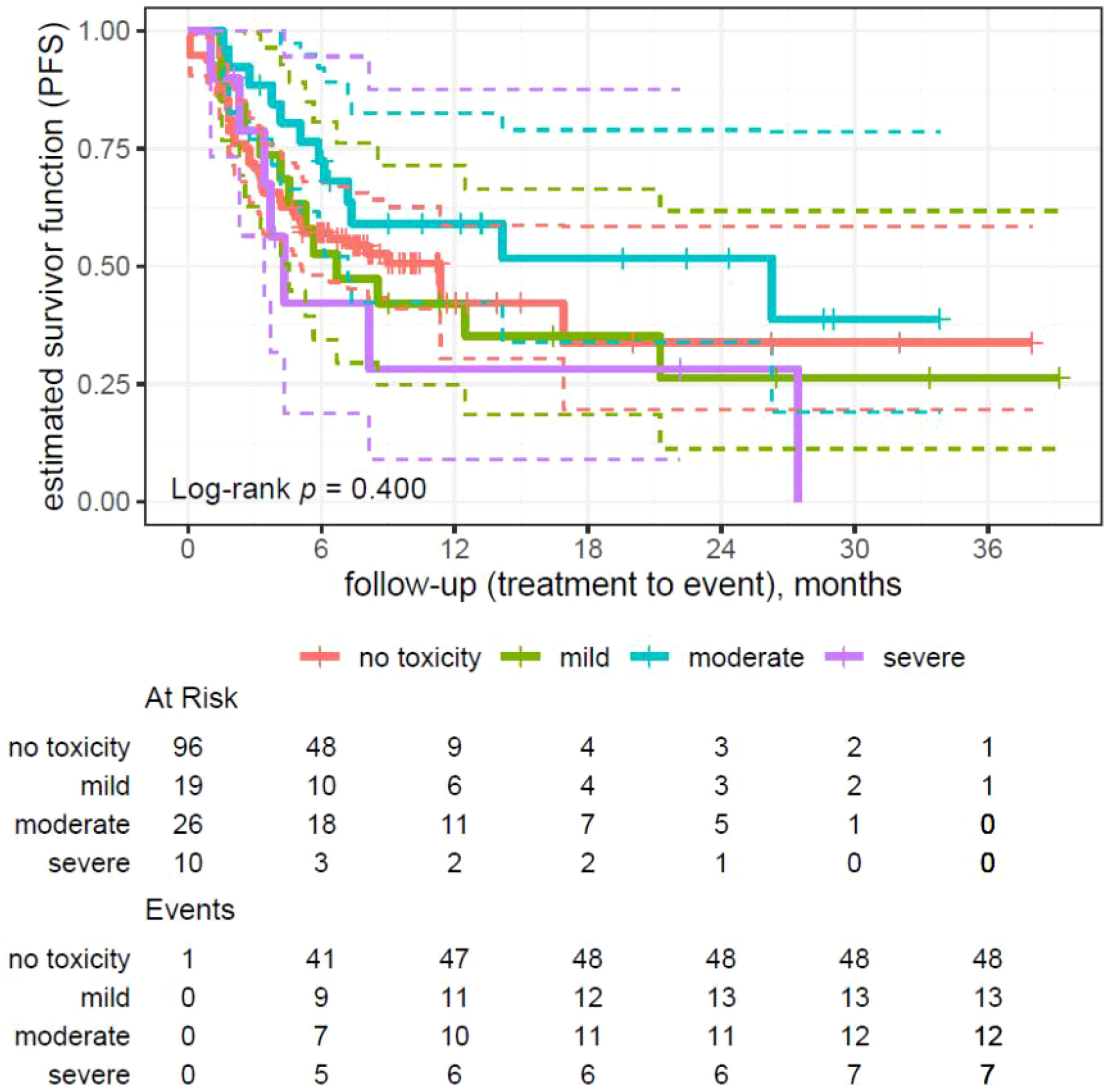
Kaplan–Meier analysis on survival with regard to disease progression divided by the severity of toxicity (no toxicity, mild, moderate, and severe toxicity).

### 3.3 Multivariable analysis

The results of the multivariate analysis using the multivariable Cox proportional hazard model evaluated the effect of different variables on OS among patients. The analysis considers variables such as age, sex, time from occurrence of treatment toxicity, number of toxicity foci, smoking factor, pack-years, and treatment line. The results are shown in **Table 2**.

**Table 2:** Cox model results (*N_obs_* = 151).

Explanatory variables	Overall survival		
	HR	CI 95%	P
Age, years	0.98	0.95–1.01	0.135
Gender (man) (with regard to women)	0.64	0.37–1.12	0.117
Toxicity occurrence time, weeks	0.97	0.95–1.00	**0.024**
Number of toxicity outbreaks	0.99	0.70–1.40	0.961
Smoking (in the past) (with regard to non-smokers)	1.12	0.52–2.41	0.765
Smoking (active smokers) (with regard to non-smokers)	1.11	0.50–2.46	0.796
Pack-years	1.01	1.00–1.03	**0.014**
Line of treatment (anti-PD1) (with regard to anti-PD-L1)	0.89	0.54–1.45	0.638

*N_obs_*, number of observations; HR, hazard ratio; CI 95%, confidence interval 95%; p, statistical test p-value.Values nearing staistical significance (p<0.05 ) are marked in bold.

The model exhibited an average level of fitting (*R^2Nagelkerke^* = 0.12) and a moderate prognostic ability (concordance = 0.628). The model did not show any phenomena of collinearity [GVIF and GVIF 1/(2df) < 2.0], and the assumption of proportional hazards for the general model has been confirmed.

*HR* in case of age factor was 0.98, which suggests a minimal decrease in death risk by 2% with each additional year of life. However, a value of p = 0.135 means that the correlation is non-significant.

Male patients appear to have lower risk of death in comparison with female patients (*HR* 0.64); however, the difference is statistically irrelevant (p = 0.117).

Each additional week of exposition to treatment toxicity was related to reduction in the risk of death for 3%. No statistically significant impact of toxicity foci on survival was found (*HR* = 0.99; p = 0.961).

PD-L1 expression was negatively correlated to the pack-year parameter, although it did not reach statistical significance (p = 0.051).

Neither ex-smokers nor active smokers did show any significant difference in death risk compared to non-smokers (p = 0.765 and p = 0.796). Concurrently, the pack-years was significantly related to death risk (*HR* = 1.01; p = 0.014).

There was no statistically significant effect of the anti-PD1 therapy in comparison with anti-PD-L1 therapy for patient survival (*HR* = 0.89; p = 0.638).

On the basis of Cox’s model results of a group of 151 patients, the impact of various factors on PFS was analyzed. The results of the coefficients of the model are reported in **Table 3**.

**Table 3:** Cox model results (*N_obs_* = 152).

Explanatory variables	Progression-free survival		
	HR	CI 95%	P
Age, years	0.97	0.95–1.00	0.063
Gender (man) (with regard to women)	0.43	0.26–0.72	**0.002**
Toxicity occurrence time, weeks	0.98	0.95–1.00	**0.033**
Number of toxicity outbreaks	0.93	0.69–1.25	0.629
Smoking (in the past) (with regard to non-smokers)	1.90	0.95–3.82	0.071
Smoking (active smokers) (with regard to non-smokers)	1.25	0.59–2.64	0.566
pack-years	1.01	1.00–1.03	**0.011**
Line of treatment (anti-PD1) (with regard to anti-PD-L1)	0.86	0.54–1.37	0.520

*N_obs_*, number of observations; HR, hazard ratio; CI 95%, confidence interval 95%; p, statistical test p-value.Values nearing staistical significance (p<0.05 ) are marked in bold.

Male patients have statistically less significant risk of progression of the disease compared to female patients (*HR* = 0.43, p = 0.002).

Each week of delay in occurrence of the toxicity correlates with reduction in the risk of progression (*HR* = 0.98, p = 0.033).

No significant effect of toxicity foci on PFS (*HR* = 0.93, p = 0.629) suggests that it is not a crucial factor affecting the progression of the disease in this cohort.

Ex-smokers demonstrate a trend toward an increased risk of the progression (*HR* = 1.90, p = 0.071). For active smokers (*HR* = 1.25, p = 0.566), no significant impact was found out.

Increase in the number of pack-years was associated with statistically significant increase in progression risk (*HR* = 1.01, p = 0.011). No statistically significant difference between PFS and anti-PD1 and anti-PD-L1 therapy has been found (*HR* = 0.86, p = 0.520).

The model exhibited an average level of fitting (*R^2Nagelkerke^* = 0.17) and a moderate prognosis ability (concordance = 0.67), which suggests that other unidentified variables might also affect the survival.

The tendency to extend the PFS with each subsequent year is observed (*HR* = 0.97). The value of *p* = 0.063 may suggest that the age has some impact on PFS.

The base model for the multivariable analysis was additionally adjusted for an organ toxicity factor. For each type of organ, a separate model was fitted; however, the analysis of adjusted Cox models on OS within the context of the occurrence of organ toxicity did not show any statistically significant correlations between the toxicity and overall survival.

**Figure 3:**
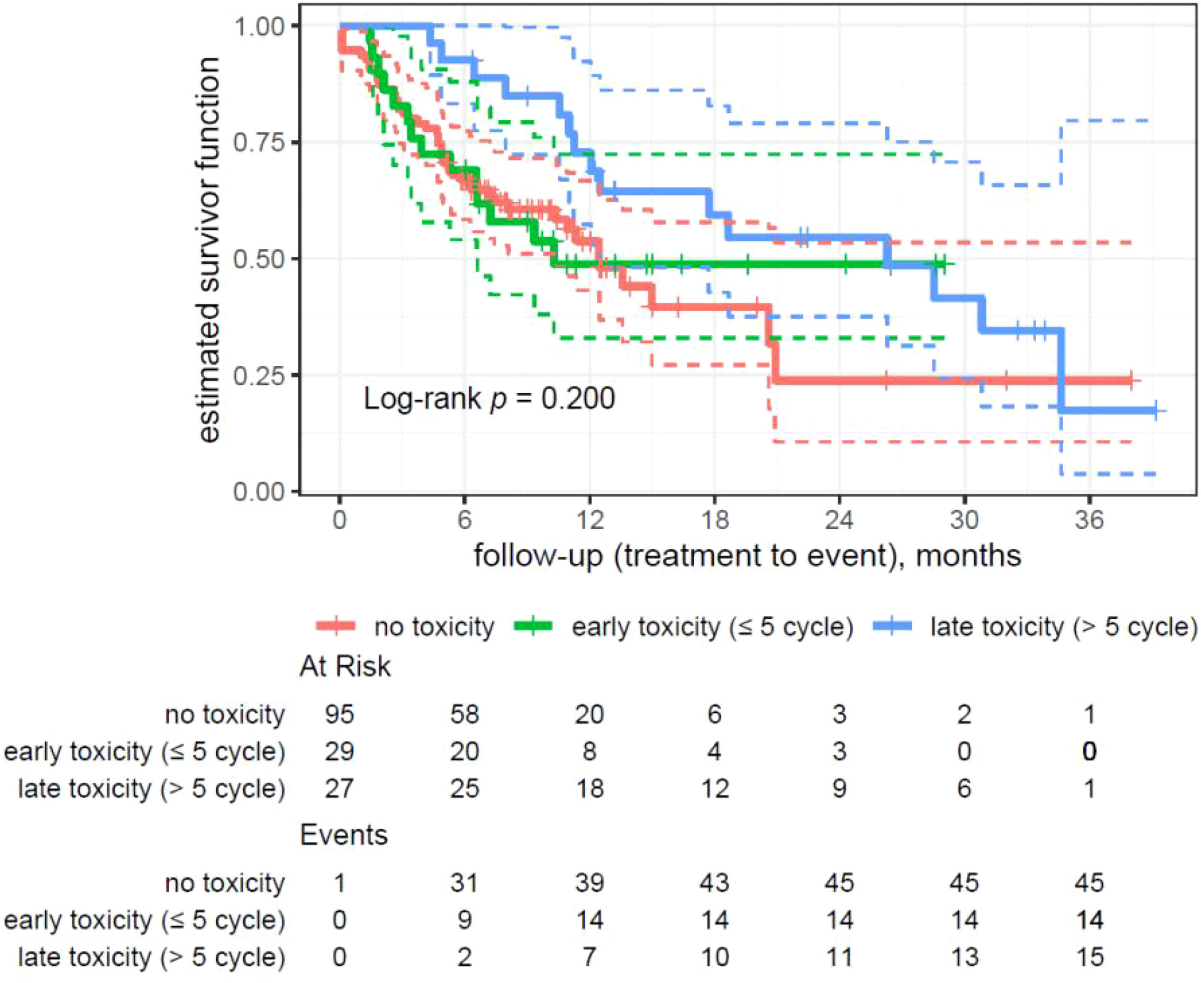
Kaplan–Meier analysis on patients’ overall survival divided by the time of toxicity onset.

### 3.4 Analysis of survival divided by the time of toxicity onset

In the conducted survival analysis among patients, differences in survival were observed depending on the moment of toxicity onset and number of treatment cycles. The analysis included three groups of patients as follows: without toxicity (95 patients, 45 events, median survival 12.5 months), early toxicity (up to 5 cycles; 29 patients, 14 events, median survival 10.3 months), and late toxicity (over 5 cycles; 27 patients, 15 events, median survival 26.3 months).

Log-rank test results did not reach the statistical significance for this analysis (p = 0.200).

**Figure 4:**
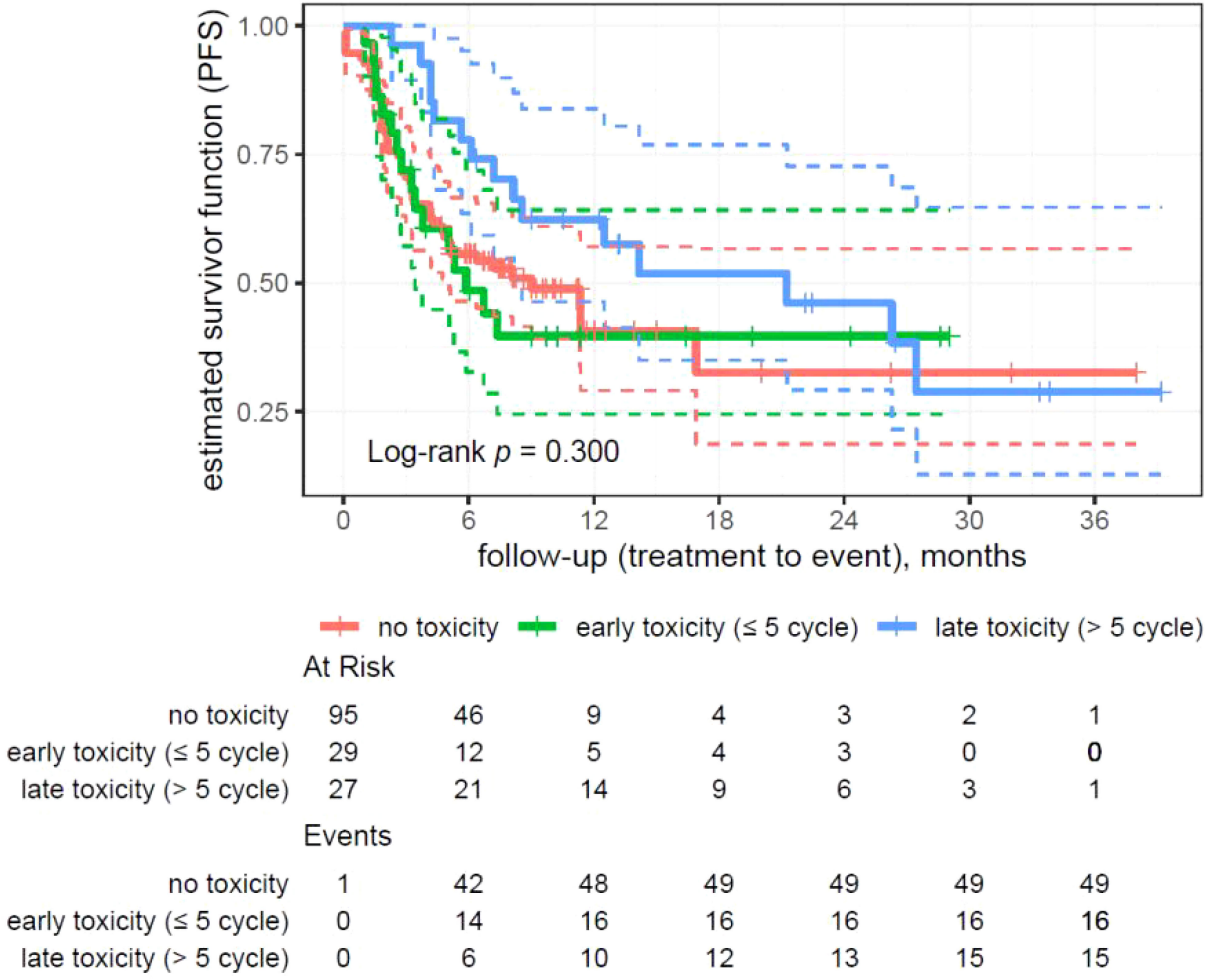
Kaplan–Meier analysis on patients’ progression-free survival divided by the time of toxicity onset.

### 3.5 Analysis of progression-free survival divided by the time of toxicity onset

The analysis showed no statistically significant differences in survival (p = 0.300) depending on the moment of the toxicity onset (**Figures 1**–**4**).

In the group without toxicity (95 patients, 49 events), the median survival was 9 months. For the early toxicity group (29 patients, 16 events), the median survival was 5.88 months. The late toxicity group (27 patients, 15 events) showed a significantly longer median survival of 21.26 months.

### 3.6 Analysis of the RECIST response pattern divided by the time of toxicity onset

The Chi-square Pearson analysis was used to compare the RECIST response according to the time of toxicity onset. Initial response and best response RECIST results were divided in two subgroups as follows: complete response (CR) and partial response (PR) versus stabilization (SD) and progression of the disease (PD). The results did not reach statistical significance (p = 0.912 for initial response and p = 0.705 for the best response).

For the initial response, 20 (25.97%) patients without toxicity experienced complete response or partial response. The early onset toxicity subgroup was represented by 7 (29.17%) patients and the late onset toxicity subgroup by 8 (29.63%) in the CR and PR groups.

For the best response, 22 (28.57%) of the patients without toxicity experienced CR or PR. CR or PR were achieved in 7 (29.17%) patients from the early onset toxicity subgroup and 10 (37.04%) from the late onset toxicity subgroup.

## 4 Discussion

The immune checkpoint inhibitors (ICI) are becoming a more prevalent systemic treatment option in a variety of neoplasm subtypes along chemotherapy and tyrosine kinase inhibitors (9, 10). Beside the advantage of the molecular mechanism of action, immunotherapy is also considered a relatively safe medicament with lower frequency of adverse effects in comparison to systemic chemotherapy (9, 11, 12). This assumption was recently put under scrutiny due to the unpredictable nature of the immune-related adverse effects (irAE) and their oftentimes extremely severe course, difficult diagnostic processes, and low treatment responses (13–15).

In the last few years, most research has focused on the association between toxicity incidence and response patterns in ICI treatment (16–23). Our study, conducted in a heterogeneous cohort with a predominant representation of NSCLC patients (78.1%), found that patients experiencing toxicity tend to have better responses—a finding consistent with the results from other studies. Improvement in survival across various malignancies with the development of immune-related adverse events has been documented in available literature (16–23).

While much of this research relates to the timing and the severity of these irAEs, the prognostic function of moderate late toxicities remains unexplored. For instance, several large systematic reviews and meta-analyses have investigated the association between irAEs and survival outcomes among patients undergoing treatment with ICIs.

However, most studies have investigated only the association between irAEs and improved overall survival, thus leaving the effect of moderate late-onset toxicities unexplored. Similarly, Han et al. and Wang et al. articles have focused primarily on severe toxicities and lethal ones, though not on those less severe adverse events that turn up later with their prognostic value (24, 25). The KEYNOTE-024 trial testing pembrolizumab in NSCLC and the CheckMate trials, including CheckMate 057 and CheckMate 017, also identified an association of severe irAEs with survival benefit but have not reported on the impact of moderate or delayed toxicities on the clinical outcome (26, 27). Likewise, the IMpower trials, dealing with atezolizumab for different types of malignancies, analyzed the effect of irAEs on survival but again did not stress how such toxicities, appearing later in treatment, could be used as a prognostic factor (28, 29). In addition, a Del Pozo et al. meta-analysis gave an overview of the overall effect of irAEs on survival but did not pay specific attention to less severe, delayed adverse events (30). Furthermore, in a research by Chen et al., the broader effects of irAEs on treatment outcomes were reviewed, but the prognostic significance of delayed or moderate toxicities was not specifically investigated (31).

Our study seeks to fill this gap by investigating the prognostic implications of moderate and late-onset irAEs providing a more nuanced understanding of their role in survival outcomes. This observation challenges some prior reports that high-grade irAEs could be beneficial and is congruent with studies pointing toward possible deleterious effects of severe toxicities. Our findings suggest the role of moderate toxicity as a marker of optimal immune activation distinguishing it from excessive immune responses that may contribute to poorer outcomes.

Moreover, our study clarifies the timing of the onset of irAEs as a predictive factor. Patients with late-onset irAEs—after five treatment cycles—exhibited significantly longer OS and PFS compared to patients who developed early-onset toxicity or no toxicity. This finding confirms emerging evidence that a delayed immune response may be more indicative of an effective, sustained antitumor activity, whereas early toxicity might reflect overly aggressive immune responses with less durable benefits. Of note, our study confirms such a trend in the real-world patient population and extends these observations to NSCLC cases, in particular, enhancing their clinical relevance.

By highlighting these distinct toxicity patterns, our study underscores the importance of considering both severity and timing when evaluating patient prognosis. These findings add to an emergent understanding of irAEs as biomarkers and suggest a need for greater consideration of treatment-related toxicities in the clinic to best optimize patient outcomes. The shorter median PFS and longer median OS of the group experiencing toxicity can indicate the possibility of the long-lasting benefits of the heightened immune activation, despite the short-lasting clinical response according to the RECIST criteria (24, 32, 33). The observed differences in median PFS might also not be related to the toxicity only but could be an effect of factors other than toxicity.

The moderate toxicity represented the highest median survival of 26 months and median progression-free survival of 26.28 months. The drastically longer PFS in comparison with the median of 6.70 months in patients with mild toxicity and median of 4.34 months in patients with severe toxicity strongly indicates moderate toxicity as an indicator of efficient treatment response. The occurrence of mild adverse effects can indicate a requirement of a dose increase to reach the clinical response.

The lowest median of survival (6.41 months) in patients with severe and very severe toxicity suggests the excessive immune response to the immunotherapy treatment in this subgroup without clinical benefit. A survival rate of 50%, with half of the patients dying in the first 6 months, marks the overstimulation of the immune system during the course of the ICI treatment presenting in the form of several adverse effects as life-threatening and highly dangerous.

Several studies found that the >3-grade irAE is advantageous for patient survival, which is contradictory to our findings (32). In the studies of Chen et al. and Wu et al., results similar to ours were obtained—mild and moderate toxicities were found beneficial in the context of survival, and >3-grade irAEs were found detrimental for the OS of the patients (32, 34). A Hussaini et al. meta-analysis associated grade 3 or 4 irAEs with increased ORR but worse OS, while a Fan et al. meta-analysis did not associate severe toxicities with a significantly favorable PFS or OS (21, 35). Ricciuti et al. found no difference in the OS and PFS for the <2- and >3-grade irAE in the small-cell lung carcinoma patients (36). Patients with mild and moderate adverse effects were found to have better OS and PFS than patients without toxicity in a study by Dey et al., which was part of the MYSTIC trial (37). It can be assumed that heightened molecular response causing adverse effects can be linked to better treatment results. The later time of onset of the irAE seems to be linked with the prolonged OS and PFS. Our research found that patients with late toxicity onset achieved over two times longer OS and PFS in comparison to the early onset toxicity and no toxicity groups. In the late toxicity onset group, OS and PFS exceeded 26 months, while early toxicity onset patients reached 5.88 months of PFS and 10.3 months of OS, and no toxicity patients reached 9 months of PFS and 12.5 months of OS. Hsiehchen et al. reported median PFS for patients with no irAE, early-onset irAE, and late-onset irAE as 2.8, 5.6, and 13.8 months, respectively (log-rank test, p < 0.010). The median OS for patients with no irAE, early-onset irAE, and late-onset irAE was 9.1, 14.2, and 30.9 months, respectively (p < 0.010). Their findings are consistent with our results and suggest late toxicity onset as an important prognostic factor of survival (38).

The correlation between each week of delay in the occurrence of the toxicity and a reduction in the risk of progression may indicate a more advantageous immune response to the treatment. The later development of toxicity indicates a gradual activation of the immune response, which lowers the possibility of the “burnout” effect in the lymphocyte population due to the overactivation. A prolonged exposure to toxicity was positively correlated with prolonged OS, which can pinpoint the toxicity occurrence as a marker of a good treatment response.

No correlation between the number of toxicity sites, type of organ-specific toxicity or implemented medicament, and PFS or OS has been found in our research. No correlation with PFS indicates that the amount of toxicity foci should not be used as a prognostic marker without any additional research.

The association between an increase in pack-years and a statistically significant increase in progression risk and death risk confirms the negative long-term smoking impact on the course of neoplastic disease. The statistical significance between pack-years and progression risk and death risk suggests that prolonged smoking experience may be associated with worse survival. Promoting smoking cessation among patients may result in PFS improvement. Pack-years may be used as a prognostic factor or to stratify risk in clinical studies.

This study brings important insights into the association of irAEs with survival outcomes in patients treated with ICIs in a real-world clinical setting from Poland. However, the findings should be interpreted with caution keeping in mind the limitations imposed by the study design.

First, this is a single-center, observational study conducted on a relatively small cohort of 151 patients with various solid tumors, with the majority having NSCLC. Although this design has its advantages regarding detailed and consistent data collection, generalization to other populations or healthcare systems may be limited. In the future, larger multi-center cohorts could improve representativeness and enable validation of these findings across broader clinical settings. Second, the inclusion of different subtypes of tumor introduces a degree of heterogeneity that reflects everyday clinical practice and may affect associations between irAEs and survival outcomes. Despite the state-of-the-art statistical methods as well as established criteria through RECIST 1.1 and CTCAE v4.0 in this study, the modest sample size may reduce the statistical power to identify certain differences in some subgroup analyses. Although this does not diminish the relevance of the findings, the fact does indicate the need for further studies to confirm such associations in larger and more homogeneous patient groups.

Moreover, whereas the present study was able to differentiate between immunotherapy- and chemotherapy-related adverse events based on their toxicity profile and timing, the complexity of treatment interactions makes it difficult to fully isolate the effects of each therapeutic approach. Some toxicities, such as fatigue or gastrointestinal symptoms, could be overlapping among treatments and thus cannot be finally attributed to either immunotherapy or chemotherapy. Furthermore, no information regarding the duration of immunotherapy was given, and any influence on the incidence and severity of irAEs or survival outcomes was not analyzed. This is a limitation of the fact that, in most patients, immunotherapy was administered as a last-line treatment leading to censoring due to either progression-free survival or mortality within a relatively short observation period. Therefore, data on the duration of exposure to immunotherapy could not be comprehensively captured. Where applicable, future studies with extended follow-up may provide longitudinal data that could help determine the relationship between treatment duration, irAEs, and patient prognosis.

Second, although the analysis in our investigation was adjusted by using a multivariable Cox proportional hazard model, residual confounding cannot be completely excluded. Several patient-dependent factors might interact, including co-morbidities, pre-treatment characteristics, and general conditions, each contributing to an impact on therapeutic outcomes and any irAE. Although our analysis was adjusted for most of the key clinical variables, some of the confounding factors—particularly those not documented routinely in the medical records—might have confounded the observed associations. Moreover, several other important confounding factors, such as genetic predisposition, molecular characteristics of the tumors, and lifestyle factors, including diet, exercise, and socioeconomic status, could not be analyzed because data were not available.

To further strengthen the validity of the findings, additional statistical techniques, such as propensity score matching or inverse probability weighting, could be employed in future analyses to minimize potential bias. Sensitivity analyses assessing the robustness of the results under different confounding assumptions may also provide deeper insights. Additionally, prospective studies with more comprehensive data collection, including biomarker profiling and detailed treatment histories, would help refine the understanding of factors influencing immunotherapy outcomes.

It is worth noting that this study was conducted retrospectively using existing medical records, which may explain why a preregistered study protocol is not mentioned. Since the data collection was retrospective, preregistration was not a common practice at the time of the study planning and the beginning of the data collection. However, to improve transparency and reproducibility, future studies could benefit from preregistering their methodology and having a clear plan for data sharing whenever possible.

In summary, this study, though limited, adds to the growing literature on ICIs and underscores the importance of monitoring toxicities for irAEs as potential biomarkers of survival outcomes. Though the analysis did not reach or consistently attain conventional statistical significance at p < 0.05, the trends in associations observed would appear clinically meaningful. Specifically, late-onset toxicity events and moderate (grade 2) irAEs may be identified as likely prognostic indicators during the course of immunotherapy treatment. These findings emphasize the need for further studies to confirm and extend these observations addressing the gaps in understanding the interplay between irAEs, treatment exposure, and patient outcomes. Such studies could ultimately enhance the prognostic and therapeutic utility of irAEs in cancer immunotherapy.

## Data Availability

The raw data supporting the conclusions of this article will be made available by the authors, without undue reservation.
