# Supplementary material for: Moderate toxicity with late onset as a good omen: association between toxicity and survival in the checkpoint inhibitor immunotherapy—a single center experience
Source: Front Immunol. 2025 Apr 28;16:1527103. doi: 10.3389/fimmu.2025.1527103 (PMC12066656; doi:10.3389/fimmu.2025.1527103)
Supplement: Supplementary file 1 [file Table1.docx]

Based on the data in table 1, adverse events like skin toxicity, thyroid toxicity, hepatotoxicity, renal toxicity, fatigue, and arthritis were relatively uncommon in both Anti-PD-L1 and Anti-PD1 treatment groups, with no significant differences between them (all p > 0.05). This indicates that both therapies are generally well-tolerated and have similar safety profiles.

**Table 1.** Frequency of adverse events associated with Anti-PD-L1 and Anti-PD1 treatments

| *Characteristic* | *N* | *Treatment* | | *p^b^* |
| --- | --- | --- | --- | --- |
|  |  | *anti-PD-L1,*  *n_1_ = 52^a^* | *anti-PD1,*  *n_2_ = 99^a^* |  |
| Skin toxicity | 151 | 3 (5.77%) | 7 (7.07%) | 1.000 |
| Thyroid toxicity | 151 | 8 (15.38%) | 10 (10.10%) | 0.341*^c^* |
| Hepatotoxicity | 151 | 4 (7.69%) | 8 (8.08%) | 1.000 |
| Renal toxicity | 151 | 2 (3.85%) | 2 (2.02%) | 0.608 |
| Fatigue toxicity | 151 | 2 (3.85%) | 1 (1.01%) | 0.273 |
| Arthritis toxicity | 151 | 0 (0%) | 2 (2.02%) | 0.545 |
| *^a^* n (%)  *^b^* Fisher’s exact test  *^c^*  Pearson’s Chi-squared test | | | | |

According to the results in Table 2, there does not appear to be a statistically significant relationship between tumor type and the number of toxicity outbreaks, as indicated by the p-value = 0.471. This indicates that, within this sample, the occurrence of toxicities was not strongly dependent on the type of tumor.

**Table 2.** Frequency of toxicity outbreaks by tumor type

| *Characteristic* | *N* | *Type of tumor* | | | | | *p^b^* |
| --- | --- | --- | --- | --- | --- | --- | --- |
|  |  | *NSCLC,*  *n_1_ = 118^a^* | *Bladder cancer,*  *n_2_ = 14^a^* | *SCLC,*  *n_3_ = 9^a^* | *Renal cancer,*  *n_4_ = 7^a^* | *Other,*  *n_5_ = 3^a^* |  |
| number of toxicity outbreaks: | 151 |  |  |  |  |  | 0.471 |
| 0 |  | 70 (59.32%) | 10 (71.43%) | 7 (77.78%) | 7(100.00%) | 1 (33.33%) |  |
| 1 |  | 36 (30.51%) | 3.00 (21.43%) | 2. (22.22%) | 0 (0%) | 2 (66.67%) |  |
| ≥ 2 |  | 12 (10.17%) | 1.00 (7.14%) | 0 (0%) | 0 (0%) | 0 (0.00%) |  |
| *^a^* *n* (%)  *^b^* Fisher’s exact test | | | | | | | |
